# Exploring Device Wear Criteria to Characterize Glucose Patterns in Prefrail Older Adults Without Diabetes

**DOI:** 10.1093/geroni/igaf122.644

**Published:** 2025-12-31

**Authors:** Amal Wanigatunga, Xiaowen Chen, Sydney Schultz, Elizabeth Selvin, Karen Bandeen-Roche, Eleanor Simonsick, Jennifer Schrack

**Affiliations:** Johns Hopkins Bloomberg School of Public Health, Baltimore, Maryland, United States; Johns Hopkins Bloomberg School of Public Health, Baltimore, Maryland, United States; Johns Hopkins Bloomberg School of Public Health, Baltimore, Maryland, United States; Johns Hopkins Bloomberg School of Public Health, Baltimore, Maryland, United States; Johns Hopkins Center on Aging and Health, Baltimore, Maryland, United States; National Institute on Aging, Baltimore, Maryland, United States; Johns Hopkins Bloomberg School of Public Health, Baltimore, Maryland, United States

## Abstract

Frail at-risk individuals may experience episodes of low blood sugar that may accelerate frailty onset. Continuous glucose monitoring (CGM) provides detailed interstitial glucose information throughout the day but device wear criteria (e.g., 24-hour wear; weekend behavior differences) to accurately estimate ambulatory glucose levels in prefrail older adults living without diabetes are not well defined. Baseline data from 37 participants enrolled in the Sedentary To Active Rising to Thrive (START) trial were examined (Dec 2023–Nov 2024). Each participant wore a CGM sensor for up to 14 days. Linear regression was used to estimate differences in mean ambulatory glucose level between: 1) wear days with and without complete data collection and 2) weekdays and weekends. Among 37 START participants, 32(86%) provided CGM data (mean age:76 years, 94%women). Mean wear period was 14-days (range: 9–15) and mean CGM glucose was 106 mg/dL (range:95–113 mg/dL). Glucose levels were lower on days with missing data (97.67 mg/dL; n = 64 days) compared to days with no missing (107.64 mg/dL; n = 393 days) (p < 0.001). Time-of-day patterns showed that mean glucose levels were lowest around 3 AM (94.5 mg/dL, SD = 27.2) and highest around 3 PM (113.4 mg/dL, SD = 16.0). Mean glucose levels were higher by 3 mg/dL on weekends compared to weekdays (p = 0.07). Findings suggest that addressing incomplete data collection would improve estimates of mean ambulatory glucose levels by capturing both fasting (early morning) and postprandial (afternoon) effects on glucose levels. Findings also suggest that at least one weekend day should be measured for valid ambulatory glucose assessment.

